# A review of progress on complement and primary membranous nephropathy

**DOI:** 10.1097/MD.0000000000038990

**Published:** 2024-07-19

**Authors:** Shanshen Yu, Jia Sun

**Affiliations:** a Department of Nephrology, First People’s Hospital of Linping District, Hangzhou, China.

**Keywords:** complement inhibitors, complement pathway, pathogenesis, primary membranous nephropathy

## Abstract

Primary membranous nephropathy (PMN) is a predominant cause of adult nephrotic syndrome, with its incidence witnessing a progressive surge over time. Approximately 35% to 47% of patients progress to renal failure within 10 years, causing a huge social burden. Within China, the proportion of PMN in primary glomerular disease exhibits a gradual ascension. Recent studies have shown that the 3 activation pathways of complement: the classical pathway, mannose-binding lectin pathway, and alternative pathway, are all involved in the pathogenesis of PMN. Despite historical limitations in detecting C1q deposits on the glomeruli of PMN in the past, recent studies have confirmed the classical pathway is implicated in patients with PMN. Considering the dysregulation of the complement system has been observed in PMN, complement inhibitors become increasingly promising. Several clinical trials are presently underway to evaluate the efficacy of complement inhibitors, such as MASP2 antagonists (OMS721), C3 and C3b antagonists (APL2), FD inhibitors (BCX9930), C3aR antagonists (SB290157 and JR14a), FB inhibitors (LNP023). This article reviews the recent research progress on the role of the complement pathway in the pathogenesis of PMN, and underscores the importance of continued research into the complement pathway and its inhibitors, which may pave the way for groundbreaking advancements in the management of PMN.

## 1. Introduction

Primary membranous nephropathy (PMN) is a predominant cause of adult nephrotic syndrome, with 35% to 47% of patients suffering from persistent massive proteinuria progressing to kidney failure within a decade.^[1]^ The incidence rate is approximately 1.2 per 100,000 individuals annually in worldwide, showing a progressive increase with advancing age. Epidemiological studies indicate variations across ethnicity and gender, with a higher prevalence observed in Caucasians, followed by Asians, and lower frequencies in Black and Hispanic populations. Additionally, the condition is more prevalent in men than in women, with a ratio of 2:1.^[2]^ In China, the renal division of Peking University First Hospital retrospectively analyzed renal biopsy data from 6049 patients with kidney disease, the proportion of PMN in primary glomerular disease increased from 16.8% (2003–2007) to 29.35% (2008–2012) in all groups of age. And young patients with PMN (14–44 years old) were significantly increasing.^[3]^ Hou’s nationwide study based on renal biopsy data from Chinese patients with kidney disease found that the frequencies of other major glomerulopathies remained stable, while the frequency of PMN increased from 12.2% to 24.9% from 2004 to 2014.^[4]^ Renal failure precipitates a heightened incidence of cardiovascular diseases,^[5,6]^ metabolic disorders,^[7]^ neurological conditions,^[8]^ and various other ailments. Consequently, an increasing number of individuals are impacted by the array of risk factors and disease burden associated with PMN.

The hallmark morphological feature of PMN is the presence of subepithelial immunoglobulin and antigen complex deposits, within the glomerular basement membrane, in which complement components are often detected.^[9,10]^ Phospholipase A2 receptor (PLA2R) and thrombospondin type-1 domain-containing protein 7A (THSD7A) are the primary target antigens in PMN, accounting for approximately 70% and 2% to 3% of all cases, respectively. The antibodies against these circulating or subepithelial antigens are predominantly of the IgG4 subclass.^[11–16]^ However, IgG4 cannot bind to C1q.^[17]^

**Figure 1.**
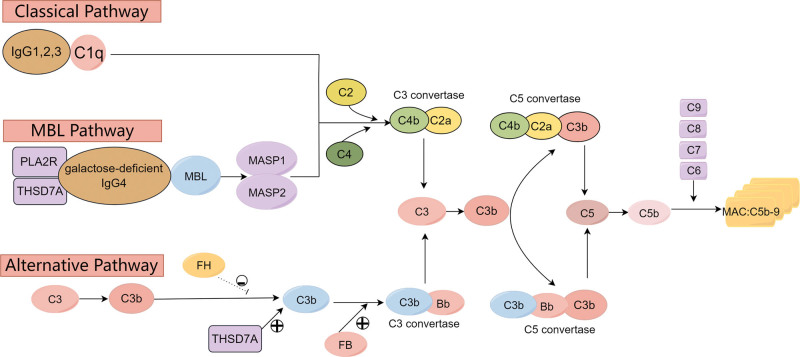
The complement system in humans. By Figdraw.

Complements are essentially a series of proteins that, upon activation, exhibit enzymatic activity and are widely distributed in fresh serum, cell membrane surfaces, and tissue fluids of humans and animals. Complement may be activated by 3 distinct pathways: first, the classical pathway is initiated by the binding of the C1 to antigen–antibody complex on the surface of a bacterial cell; second, the mannose-binding lectin (MBL) pathway is activated by the attachment of MBL or ficolins to pathogen-associated molecular patterns on the bacterial surface; third, the alternative pathway is constantly activated by the spontaneous hydrolysis of C3 (Fig. 1).^[18,19]^ Inherent regulators include C1-Inhibitor, Factor I, Factor H, C4b-binding protein, etc.^[20]^

Recent researches show that complement is involved in various renal diseases, including antibody-mediated glomerulopathies, such as IgA nephropathy, MN, immune complex-mediated membranoproliferative glomerulonephritis, anti-GBM glomerulonephritis, and non-antibody-mediated glomerulopathies, like atypical hemolytic uremic syndrome and C3 glomerulopathy.^[21–24]^ This article reviews the research progress on the mechanism of complement pathways in the pathogenesis of PMN.

## 2. MBL pathway and PMN

PLA2R and THSD7A were identified as intrinsic antigens in PMN. Hayashi et al revealed that the staining intensity and prevalence of MBL deposits were significantly higher in both PLA2R-related and THSD7A-related group, which also correlated with the IgG4 staining intensity.^[25]^ Haddad et al found that there was a correlation between elevated levels of galactose-deficient IgG4 and anti-PLA2R titers. Furthermore, they demonstrated that anti-PLA2R IgG4 autoantibodies could activate the MBL pathway in a glycosylation-dependent manner, leading to podocytes injury.^[26]^ Li et al found that in the PLA2R-associated MN, the expression and intensity of glomerular MBL deposition was higher in the nephrotic syndrome group compared to the remission group, indicating that MBL deposition was correlated with non-remission.^[27]^ While Zhang et al found that PMN patients with glomerular MBL activation can easily reach proteinuria incomplete remission (urinary protein 0.3–1.0 g/day). 79.1% of patients with PMN showed MBL deposition, co-localizing with PLA2R in immune deposits.^[28]^ Wang et al found that human anti-THSD7A antibody promoted serum MBL-associated serine protease-1 (MASP-1), MBL-associated serine protease-2 (MASP-2), MBL, C3a and C5a expression reflecting the anti-THSD7A antibody involved in MBL pathway in mice.^[29]^

In 1979, British scholars published the first report linking HLA-DR3 to PMN.^[30]^ Since then, increasing attention has been directed towards genetics and bioinformatics in understanding this connection.^[31–34]^ A higher frequency of polymorphisms in exon 1 of the MBL2 gene was observed in patients with MN, compared to healthy counterparts, which may be linked to the MBL pathway activation.^[35]^

## 3. Alternative pathway and PMN

In MN, various complement proteins and regulatory proteins are observed, including complement proteins C3, C4, C5, C6, C7, C8, C9, as well as complement regulating proteins such as FH, FHR5, FHR1, FHR2, FHR3, clusterin, vitronectin, and CFB.^[36]^ An in vitro experiment showed that C3b activated by THSD7A immune complexes was completely abolished in FB-depleted sera, partially inhibited in C4-depleted sera, and unaffected in C1q-depleted sera. This result indicates that the alternative pathway is essential to initiate complement activation by IgG4-dominant THSD7A immune complexes.^[37]^ Using wild-type and factor B-null mouse models, Luo et al found that there was no C3c and C5b-9 deposition, nor albuminuria in the factor B-null model, contrast to their wild-type counterparts. This finding indicates that the alternative pathway is essential for the pathogenesis of MN.^[38]^

Complement factor H (CFH) is a key regulator of the alternative pathway. In 2018, a case was reported of a patient with PLA2R-related PMN who developed increasing levels of anti-CFH autoantibodies, subsequently leading to impaired renal function.^[39]^ A retrospective study in Japanese patients with PMN showed that the level of anti-CFH antibody was higher in patients with PMN than in healthy counterparts, suggesting anti-CFH antibody titer was identified as an independent risk factor for renal dysfunction in patients with PMN.^[40]^ While, there was also evidence indicating that anti-CFH antibodies did not lead to hyperactivation of the alternative pathway or accelerate disease progression in MN.^[41,42]^

## 4. Classical pathway and PMN

In the past, C1q deposits generated in the classical pathway usually did not exhibit on the glomerulus of PMN patients, and IgG4 cannot bind to C1q. So it was previously believed that classical pathway were not involved in the pathogenesis of PMN.^[26,40,43]^ PMN is characterized by IgG4-rich deposits, whereas secondary forms, especially lupus MN, may show predominant IgG1, IgG2, IgG3 deposits and the additional presence of C1q, implicating the classic pathway activation in Secondary MN.^[44]^

However, exceptions exist, as approximately 5% to 7% of patients with PLA2R-related MN lack IgG4 subclass antibodies.^[45]^ Seifert et al identified the presence of subepithelial C1q co-localized with IgG, and IgG-C1q proximity ligation in MN cases, indicating the activation of the classical pathway.^[46]^ Zhang et al showed that 22.9% of patients with PMN exhibited weak C1q deposits along the glomerular capillary walls, with MN stage III being notably more prevalent in these cases. The findings indicated the classical pathway activation, but may not play an essential role in mediating the kidney injury.^[47]^ On the contrary, Huang et al found that in stage I of PMN, glomerular deposits were IgG1 predominant in most cases; then in later stages, IgG4 deposits were dominant. The intensity of IgG4 and C1q staining showed an inverse relationship, inferring the classical pathway may be involved in the early stage of PMN.^[48]^ C1q depositions were more commonly observed in pediatric patients with PMN, especially primary segmental MN.^[49]^

The correlations between neurological and renal diseases have been increasingly described and studied, and often share vascular deterioration.^[8]^ Ischemic stroke in patients with PLA2R positive PMN is unusual. A previous study in Chinese people suggested that a high level of anti-PLA2R antibodies may serve as an unfavorable predictor of ischemic stroke, which in turn implying a worse prognosis for kidney disease.^[50]^ The study revealed elevated serum C1q levels in ischemic stroke patients.^[51]^ Furthermore, ischemic neurons notably expressed C1q post-stroke, potentially facilitating the attack or clearance of damaged neurons and cellular debris.^[52]^ The intricate relationship between PMN, classical complement pathways, and cerebrovascular diseases warrants comprehensive exploration.

## 5. Complement inhibitors undergoing development in PMN

Considering the dysregulation of the complement system has been observed in a wide spectrum of renal diseases, complement inhibitors become increasingly promising, and are currently being evaluated in clinical trials. Three complement inhibitors OMS721, which targets the lectin pathway protease MASP-2; APL2, which binds to C3 and C3b; and BCX9930, which targets the Factor D of alternative pathway, are all in phase II clinical trials of PMN. However, a study on the C5 inhibitor eculizumab has never been published due to negative results.^[53–56]^ Seifert et al elucidated that C3 could mediate podocyte injury and proteinuria. Furthermore, they demonstrated that C3-targeted therapy, initiated post-onset of proteinuria, could effectively eliminate glomerular C3 and the C5b-9 complex, thereby mitigating the disease.^[46]^ A Chinese study demonstrated that patients with PMN exhibited significantly elevated levels of serum C3a, along with overexpression of C3aR in podocytes, both of which were correlated with the clinical severity of the disease. C3aR antagonists, SB290157 and JR14a, could block these effects, attenuating proteinuria, electron-dense deposition, foot process width and glomerular basement membrane thickening, also alleviating plasma C3a levels and overexpression of C3aR.^[57]^ An experimental rat model of MN demonstrated that the disease could be ameliorated by blocking the alternative pathway with LNP023, a highly potent FB inhibitor, as evidenced by a marked reduction in proteinuria, improvement of overall histopathology score, and attenuation of glomerulopathy.^[58]^

## 6. Conclusions

In recent years, significant advancements have been made in understanding the inter-relationship between target antigens, pathogenic antibodies, and the dysregulation of complement pathways in PMN. The classical pathway, MBL pathway and alternative pathway were all activated in PMN. Complement inhibitors become increasingly promising in PMN. Ongoing clinical trials of complement inhibitors, such as MASP2 antagonists (OMS721), C3 and C3b antagonists (APL2), FD inhibitors (BCX9930), C3aR antagonists (SB290157 and JR14a), and FB inhibitors (LNP023), hold great potential. The eagerly awaited results of these trials may offer new therapeutic avenues, mitigating the progression to renal failure and significantly improving patient outcomes. The continued exploration of the complement system in PMN is crucial and may lead to groundbreaking advancements in disease management and patient care.

## Author contributions

**Conceptualization:** Shanshen Yu.

**Data curation:** Jia Sun.

**Funding acquisition:** Shanshen Yu.

**Supervision:** Shanshen Yu.

**Visualization:** Shanshen Yu.

**Writing – original draft:** Shanshen Yu.

**Writing – review & editing:** Shanshen Yu.
